# Enhancing the Usability of Brain-Computer Interface Systems

**DOI:** 10.1155/2019/5427154

**Published:** 2019-06-16

**Authors:** Hyun Jae Baek, Min Hye Chang, Jeong Heo, Kwang Suk Park

**Affiliations:** ^1^Department of Medical and Mechatronics Engineering, Soonchunhyang University, Asan, Republic of Korea; ^2^Korea Electrotechnology Research Institute (KERI), Ansan, Republic of Korea; ^3^Artificial Intelligence Laboratory, Software Center, LG Electronics, Seoul, Republic of Korea; ^4^Department of Biomedical Engineering, College of Medicine, Seoul National University, Seoul, Republic of Korea

## Abstract

Brain-computer interfaces (BCIs) aim to enable people to interact with the external world through an alternative, nonmuscular communication channel that uses brain signal responses to complete specific cognitive tasks. BCIs have been growing rapidly during the past few years, with most of the BCI research focusing on system performance, such as improving accuracy or information transfer rate. Despite these advances, BCI research and development is still in its infancy and requires further consideration to significantly affect human experience in most real-world environments. This paper reviews the most recent studies and findings about ergonomic issues in BCIs. We review dry electrodes that can be used to detect brain signals with high enough quality to apply in BCIs and discuss their advantages, disadvantages, and performance. Also, an overview is provided of the wide range of recent efforts to create new interface designs that do not induce fatigue or discomfort during everyday, long-term use. The basic principles of each technique are described, along with examples of current applications in BCI research. Finally, we demonstrate a user-friendly interface paradigm that uses dry capacitive electrodes that do not require any preparation procedure for EEG signal acquisition. We explore the capacitively measured steady-state visual evoked potential (SSVEP) response to an amplitude-modulated visual stimulus and the auditory steady-state response (ASSR) to an auditory stimulus modulated by familiar natural sounds to verify their availability for BCI. We report the first results of an online demonstration that adopted this ergonomic approach to evaluating BCI applications. We expect BCI to become a routine clinical, assistive, and commercial tool through advanced EEG monitoring techniques and innovative interface designs.

## 1. Introduction

An episode of the TV series *Star Trek* first shown in 1966 describes a man, Captain Pike, who suffers from locked-in syndrome. He is cognitively intact, but his body is paralyzed, leaving him confined to a wheelchair controlled by his brain wave responses to flashing lights that indicate “yes” and “no.” Dr. Octopus, the villain in the movie *Spiderman* 2, which premiered in 2004, used brain-controlled equipment to operate four mechanical arms designed with tentacle-like flexibility and gripping capabilities. That machine was controlled by thought through an interface at the spinal cord level. Jake Sully, the protagonist of *Avatar*, which premiered in 2009, was in a wheelchair in his human body, but he could walk, run, and jump in the form of his avatar, a 10-foot alien. All those characters are fictional representations of the ultimate goal of research into brain-computer interfaces (BCIs), sometimes called brain-machine interfaces. Human physical interactions, including communication, require motor control processes that use neuromuscular systems to activate and coordinate muscle movements. An individual's intent triggers the activation of a specific brain area, sending signals through the peripheral nervous system to muscles that perform the movement necessary to complete the intended task. During the past several decades, much research has been done to bypass nonfunctional muscular control channels, attempting to translate a person's intent by analyzing brain signals to empower patients with cognitive or sensorimotor problems [1–4]. The primary goal of BCI technology is to provide communication capabilities that can improve the quality of life for people severely disabled by neuromuscular impairments including amyotrophic lateral sclerosis (ALS), brainstem stroke, cerebral palsy, or spinal cord injury. For example, Sellers and Donchin [5] and Nijboer et al. [6] have reported that ALS patients can communicate using a P300 speller. Pfurtscheller et al. trained a quadriplegic patient to control an electrically driven hand orthosis using EEG signals recorded through the sensorimotor cortex [7]. In a case study performed by Leeb et al., the spinal-cord-injured subject was able to generate bursts of beta oscillations in an EEG by imagining the movements of his paralyzed feet, and those oscillations were used for self-paced BCI control of a wheelchair in virtual reality [8]. Rapid progress toward those goals is being made by many BCI research groups, allowing BCI research to expand from communication to rehabilitation medicine through neurofeedback for stroke, autism, attention deficit hyperactivity disorder, and other disorders [9–12]. However, although significant advancement has been made in BCI research, much more progress must be made before BCI can have a significant effect in real-world environments [13–15]. In particular, previous research has generally lacked understanding of (or perhaps simply failed to pay attention to) ergonomics issues, such as aesthetic designs, user-friendly interaction methods, and usability. Progress in BCI research has mainly been made by enhancing BCI performance with respect to accuracy, information transfer rate (ITR), or the number of possible selections. Thus, most of the BCI literature focuses on advanced signal processing methods or new applications or task designs, and the applications controlled by state-of-the-art interfaces have largely been intended for research-oriented environments. Most current BCI techniques face two major challenges that prevent them from being useful in real-world settings.

1. Advanced monitoring of brain activity: BCI research has used various neural signals that can be recorded noninvasively, such as electroencephalograms (EEGs), magnetoencephalograms, functional magnetic resonance imaging, and near-infrared spectroscopy. Of those, the EEG is the most commonly used method because it is noninvasive, offers high temporal resolution and portability, and has a reasonable cost. Conventional wet Ag/AgCl electrodes are used most frequently to measure EEG signals because their characteristics have been widely studied and discussed in detail [16–18]. The quality of EEG signals measured using those electrodes with skin preparation techniques and conductive gels is excellent. However, the requirement for wet electrodes greatly limits the applicability of BCIs in everyday use [19–22]. For practical use, BCIs should avoid typical EEG preparation procedures, including head measurement for accurate electrode placement and scalp preparation that requires an abrasive paste or gel to reduce skin-electrode impedance. Furthermore, electrodes for daily BCI use should not make users feel uncomfortable or look unusual. It should be possible to take EEG signal measurements from simple caps that contain EEG electrodes in appropriate montages without the need to remove or specially treat scalp hair.
2. Interface paradigm design: In a state-of-the-art BCI system, the control command, such as moving a cursor, is typically assigned to a specific mental state. The subject needs to perform a specific mental task to encode the desired control command through an attention shift or other voluntary regulation of the EEG. Currently, several types of EEG signals have been recognized: sensorimotor rhythm (also known as the *μ*/*β* rhythm), slow cortical potential, steady-state visual evoked potential (SSVEP), and event-related P300 potential. According to the literature survey performed by Hwang et al. [23], the most frequently used BCI paradigm is the motor imagery method. During actual or imagined movement, slow negative voltage shifts occur in EEGs recorded over the sensorimotor cortex, and the intention of a subject can be detected using that voltage shift. Recent motor imagery-based BCIs have used rhythmic EEG activity called event-related desynchronization/synchronization. During actual or imagined movement, event-related desynchronization occurs predominantly over the contralateral brain motor area, making it useful as a signal for a BCI system. Those methods do not require external stimuli to induce the desired EEG response. However, some drawbacks, including poor multidimensional control, high probability of error, and a need for long-term training, have led to a decrease in the use of motor imagery from 2007 to 2011. During that time, the proportion of research into the visual P300 and SSVEP paradigms has increased significantly. P300 and SSVEP require little training time and show a relatively high information transfer rate compared with other BCI paradigms. However, the requirement for a visual stimulus, such as flashing digits, letters, or other symbols that a user has to watch, has limited the flexibility, accessibility, and usability of those BCIs in real-world applications. Ideally, human-computer interactions should be free from sudden changes in luminance or high-contrast visual objects to minimize user visual fatigue and discomfort, especially for long-term use.

In 2012, Liao et al. proposed the concept of augmented BCI (ABCI) that would be appropriate for everyday use [24]. ABCI aims to expand the application of BCI technologies from their current laboratory or clinical settings to normal daily life by making them function while people move and interact with their environment. According to Liao et al.'s definition, ABCI includes nonintrusive and rapid-setup EEG solutions that require no or minimal training and thereby provide stability, robustness, comfort, and longevity for accurate long-term data collection. It also includes advanced algorithmic approaches to analyzing and interpreting brain signals measured under noisy, real-world conditions. In this review, we emphasize new paradigm designs that fit the scope of ABCI and will not induce fatigue or discomfort during everyday, long-term use. First, we survey BCI articles that discuss ABCI research. Then, we demonstrate SSVEP-based and auditory steady-state response- (ASSR-) based BCIs that use recently developed polymer foam-based capacitively coupled EEG electrodes. Our demonstration study was approved by the Institutional Review Board of the Seoul National University College of Medicine, Seoul, Korea.

## 2. Advanced EEG Monitor

Technological advancements have greatly simplified the measurement and assessment of biopotential signals, particularly electrocardiograms. However, the sites for EEG electrodes are mostly covered with hair, and EEG signals are weaker than those used in other bio-potential measurement tools, which makes the use of dry electrodes in EEG difficult. Most dry EEG electrodes make signal measurements by penetrating the outermost layer of the skin, the stratum corneum, using microelectromechanical or carbon nanotube (CNT) techniques [25–28]. However, those types of dry electrodes are somewhat invasive, and electrodes that penetrate tissue always carry the risk of infection. In addition, those techniques do not allow EEG signals to be recorded through hair, and therefore, hair and scalp preparation is still required.

**Figure 1:**
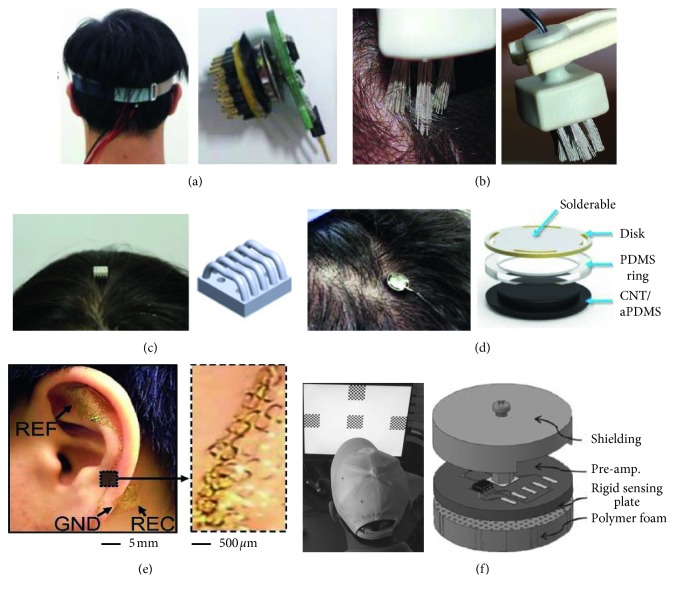
Several types of EEG electrodes: (a) active comb-shaped (electrode finger type) electrode [29], (b) bristle electrode [30], (c) reverse-curve-arch-shaped electrode [31], (d) carbon nanotube-based capacitive electrode [32], (e) epidermal electrode on the auricle [33], and (f) foam-surfaced capacitive electrode for use over hair [34, 35].

Another approach is to use electrode-finger-based sensors for EEG acquisition over hair (Figure 1(a)) [29, 36, 37]. This kind of electrode offers high geometric conformity between the electrode fingers and the irregular scalp surface, thus maintaining low electrode impedance. Additionally, the flexible substrate in which the spring probes are inserted permits the attachment of the sensor to the scalp without pain when force is applied. Similarly, a flexible, low-cost electrode about the size of a toothbrush made of polymer silver-coated bristles was suggested in 2011 (Figure 1(b)) and showed BCI feasibility using the motor imagery and oddball paradigms [30]. The main drawback of those electrodes is that they still require skin preparation to ensure contact between the finger-electrode and the scalp. Also, some participants reported prickling and other uncomfortable sensations.  Figure 1(c) shows a reverse-curve-arch-shaped dry EEG electrode 3D-printed from sterling silver to increase the skin-electrode contact area over hair [31]. The curvature of the arches was designed to match the curvature of the scalp to maximize the contact area and disperse the pressure, thereby lessening the pain induced by conventional finger-type EEG electrodes.

Lee et al. proposed an electrode composed of a CNT/aPDMS (adhesive polydimethylsiloxane) nanocomposite material (Figure 1(d)) [32]. This electrode is elastic, highly conductive, self-adhesive, and able to make conformational contact with and attachment to a hairy scalp. Hundreds of conductive cylindrical pillars coated with a Parylene C insulation layer were fabricated on a conductive disk. The CNT/aPDMS layer was then attached to the disk to transmit the EEG signal to the pillar and eliminate the air gap caused by hair, thereby maximizing the capacitance between the electrode and the scalp. The top of the disk was designed to be solderable, enabling the electrode to be connected to a variety of commercial EEG acquisition systems. Even though positive results have been published, these electrodes still have some drawbacks: they require multistep preparations and obtrusive wiring interfaces.

Epidermal electronics is an emerging class of integrated electronic systems that achieve thicknesses, effective elastic moduli, bending stiffness, and areal mass densities that match the skin [38, 39]. This technology has been proved feasible for many medical applications, such as monitoring vital signs. In particular, Norton et al. demonstrated an ultrathin, foldable neural electrode platform that could measure EEG signals from the surfaces of the outer ear (the auricle) and adjacent regions (the mastoid), as shown in Figure 1(e) [33]. The epidermal EEG electrode offers conformal contact and adequate adhesion based on van der Waals interactions alone in a manner that is mechanically unnoticeable to the user. It stayed well attached to skin with a complex surface topology (the auricle and mastoid) for more than 2 weeks, offering continuous monitoring without frequent removal or reapplication. That demonstration also showed BCI capabilities using SSVEP and P300. The electrode is soft, stretchable, and lightweight, so it can offer long-term, high-fidelity recording of EEG signals in daily life without user discomfort. However, the current sensing platform requires careful device mounting and complete dissolution of the polymer backing to allow successful EEG acquisition. Further progress in this area should consider people who do not want stick on temporary tattoos on their bodies.

A capacitive noncontact electrode that can measure EEG signals despite insulation by hair is another promising alternative to conventional wet electrodes for next-generation EEG measurement. An EEG could be measured through hair using displacement current with capacitive coupling. According to an electronic model of capacitive EEG measurement described in various studies, the capacitive measurement of EEG signals is characterized by very high electrode impedance created by the insulating effect of hair between the sensor and the scalp. Because the electrode impedance in a capacitive electrode is much higher than that in conventional wet electrodes, a high-input impedance amplifier is used as the active electrode in each sensor to convert the displacement current into voltage. Designing such an input impedance amplifier is a major challenge. Chi et al. developed a capacitive electrode that uses a custom integrated, high-impedance, low noise analog front-end [40]. The amplifier fully bootstraps both the internal and external parasitic impedances by including a low-leakage on-chip biasing network without external resistors that operates from hundreds of giga ohms to tera ohms. They also demonstrated an SSVEP-based phone dialing application that used the developed capacitive EEG electrode with two subjects [41]. The result was feasible, but the average ITR was lower than that with conventional wet electrodes. Around the same time, Baek et al. suggested a polymer foam-based capacitive EEG electrode that combines an electrode face with polymer foam adaptive to head topography (Figure 1(f)) [34]. The rigid surface of previous conventional capacitive electrodes cannot adapt to head curvature and the hair-made irregular surface that produces hundreds of micrometer-wide air gaps between the scalp and the electrode face. The use of foam minimized the loss of electrode contact area and generated increased contact impedance. The soft foam used in Baek's study enabled intimate electrode contact on the hairy scalp topography, thereby increasing the effective contact area. In addition, the foam-surfaced electrode maintained stable contact during motion, minimizing how much the electrode slid over the hair through its cushioning effect and textures. This electrode also showed BCI feasibility under SSVEP and ASSR [35]. The current capacitive electrode designs involve bulkier structures than the wet EEG electrodes widely used both clinically and academically. To translate laboratory nonclinical work into real-world clinical applications, studies should consider methodologies that maximize coupling capacitance while using small capacitive electrodes because the size of an EEG electrode is directly related to the spatial resolution of the EEG.  Table 1 summarizes the features, strengths, and drawbacks of the dry EEG electrodes reviewed above.

New EEG electrodes will improve the state of the art and increase practicality, efficacy, and ease of use. Aesthetic perspectives also should be considered. For applications outside of hospital and laboratory environments, EEG measuring devices should not make users look strange.

## 3. Interface Paradigm Design

**Figure 2:**
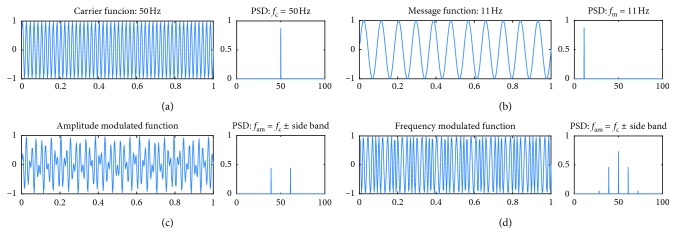
Example of time series simulation waves and their spectra: (a) a sinusoidal carrier wave at 50 Hz, (b) a message wave at 11 Hz, and (c) an amplitude-modulated signal, and (d) a frequency-modulated signal. Note that the spectra for the modulated signals show peaks at the sidebands at the carrier frequency ± message frequency.

The SSVEP approach has been widely used in BCI systems because it is simple and precise about the stimulus frequency. SSVEP-based BCIs provide high ITRs with minimal user training and require fewer EEG channels than other techniques. However, they can be annoying or fatiguing for some users, which makes them impractical. Some efforts to alleviate visual fatigue have created higher-frequency SSVEP-based BCIs that use a stimulus frequency of more than 35 Hz to decrease the feeling of flickering. However, more people were unable to complete BCI tasks with high-frequency SSVEPs than with low-frequency SSVEPs [42–45]. In 2014, Chang et al. proposed amplitude-modulated (AM) visual stimuli to elicit integer and noninteger harmonic SSVEPs, including both low- and high-frequency bands [46]. As shown in Figure 2(c), the AM signal was presented as an amplitude variation in a carrier signal, as described in equation (1). Different combinations of carrier and modulation frequencies elicited different harmonic frequencies from the low- to high-frequency range, while the visual stimulus actually flickered at a high frequency. Their experiments demonstrated that AM SSVEP with an optimized combination of harmonic frequencies performed as well as a typical SSVEP. Subject evaluations indicated reduced eye fatigue and less flickering sensation. Similarly, in 2015, Dreyer and Herrmann showed frequency-modulated (FM) visual stimuli for SSVEP BCI [47]. The FM signal, simply expressed in equation (2), encodes stimulation in a carrier wave by varying the instantaneous frequency of the wave (Figure 2(d)). This contrasts with AM, which varies the amplitude of the carrier wave while the frequency remains constant. In their experiment, they used different FM stimulation combinations that all had their lower sidebands at 10 Hz, allowing them to use FM stimulation to evoke a 10 Hz SSVEP peak without the conscious perception of a 10 Hz flicker. FM-SSVEPs with different carrier and modulation frequencies can reliably be evoked with spectral peaks at the low FM sideband of 10 Hz. Subjective perceptibility ratings for flickering decreased as the FM carrier frequencies increased, while the peak amplitude and signal-to-noise ratio remained the same.

(1) $$\begin{array}{c} S_{\text{AM}}=-\frac{1}{2}\left[\cos\left(2\pi \left(f_{\text{c}}+f_{\text{m}}\right)t\right)+\cos\left(2\pi \left(f_{\text{c}}-f_{\text{m}}\right)t\right)\right], \end{array}$$

(2) $$\begin{array}{c} S_{\text{FM}}=\sin\left(2\pi f_{\text{c}}t+M\sin\left(2\pi f_{\text{m}}t\right)\right). \end{array}$$

Several recent studies have proposed a half-field stimulation pattern based on the brain mechanism of visual selective attention [48–51]. The user is expected to concentrate their eyes on a fixation point in the middle of two flickers modulated to specific frequencies. Considering the role of the optic chiasm, SSVEP was found to be strongly modulated by spatial selective attention. The two stimulus frequency components could be extracted from the contralateral occipital regions because SSVEP enlarges substantially in response to a flickering stimulus at an attended versus an unattended location. Yan et al. showed results from a multicommand, half-field SSVEP BCI. The visual display contained 9 visual targets with 18 flickers that were realized by combining 3 stimulation frequencies. Test results from 8 subjects showed an average classification accuracy of 75.8% [50]. Punsawad and Wongsawat also showed a half-field SSVEP BCI, but they used only one visual stimulus with two black boxes on both sides of the flicker to generate 4 commands by focusing to either side of the black box or on the flicker or by closing their eyes. In this study, the average classification accuracy was approximately 77% for 4 volunteers [51].

**Figure 3:**
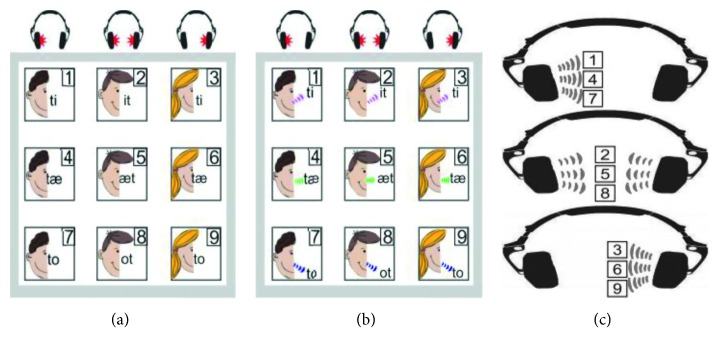
Graphical representation of the auditory stimuli set composed of spoken and sung syllables proposed by Höhne et al. [53].

Many researchers have tried to use auditory signals instead of visual ones, especially for severely impaired users who have difficulty in controlling their voluntary extraocular movements or fixing their gaze on specific visual stimuli. As with the visual paradigm, auditory BCIs have also used unpleasant auditory stimuli that might be annoying or fatiguing to users. Research using spoken or sung syllables or polyphonic musical or even natural sounds has shown that such stimuli are perceived as more pleasant, and in some cases, they even lead to better classification performance. Lopez-Gordo et al. presented a novel fully auditory BCI based on a dichotic listening paradigm using human voices (two distinct streams of letters or sentences) as the stimulus [52]. The stimuli were read out simultaneously to the subjects for binary classification using selective attention. Prior to stimulation onset, an auditory question was read to the subject by an experimenter, followed by a beep sound indicating the beginning of the dichotic listening task. The subjects were asked to pay attention to the stimulus delivered to the left ear if the correct answer to the auditory question given before the beep sound was “yes/true” and to the stimulus delivered to the right ear if the answer was “no/false.” The classification was established by recognition of the early component of human auditory evoked potentials, namely, the N1 and P2. Based on experimental results with 12 participants, they concluded that an auditory BCI evoked by natural speech showed promising results in terms of performance, usability, training, and cognitive effort. Höhne et al. explored spoken and sung syllables as the auditory stimuli [53]. Syllables that contained the vowels “i,” “æ,” or “o” were recorded by three different speakers and presented from the left ear only, right ear only, or both ears. This made a 3 × 3 matrix auditory paradigm, and a nine-class auditory BCI experiment was conducted with 9 healthy subjects, as described in Figure 3. Compared with a conventional, artificially generated monotone, the spoken or sung stimuli were expected to contain richer internal classification cues, including harmonics, pitch, and voice characteristics, as well as higher levels of variance and jitter in the auditory event-related potential (ERP) responses. The experimental results showed better classification performance when using the syllables than that when using the monotone and an increase in the subjective ergonomic ratings. Treder et al. used polyphonic music as the auditory stimulus in a multistreamed oddball paradigm [54]. The subject was asked to shift their selective attention to one of three different musical instruments, bass, drums, or keyboard, in a musical audio clip. The attended instrument could be classified with an average accuracy of 91% among 11 participants. Heo et al. proposed a novel stimulation method to minimize auditory stress by replacing the monotone carrier with familiar music and natural sounds in an ASSR-based paradigm [55]. The sounds of a violin and a piano were used as music carriers, and a cicada singing and water streaming were used as natural sounds. The violin and water streaming sounds were amplitude-modulated with a 38 Hz message frequency, and the piano and cicada singing sounds were modulated with a 42 Hz message frequency. The experimental results with six healthy subjects demonstrated that a high ergonomic rating could be acquired while maintaining high average binary classification accuracies, 74%, 89.67%, and 87.67%, for the monotone, music, and natural sound carrier waves, respectively. In conclusion, the use of pleasant sounds including a human voice or polyphonic musical or natural sounds instead of the conventional unpleasant monotone beep sound needs to be considered because the sounds can affect the level user interest and cognitive effort, even leading to improved classification performance.

## 4. Experiments and Results

In the current experiments, we used dry EEG electrodes [34] with BCIs using [46] SSVEP and [55] ASSR. Three healthy subjects (all males aged 26 to 30) who had no history of neurological disease and no neuropathological abnormalities agreed to carry out capacitive EEG measurements under new ABCI paradigms. The EEG data were recorded using foam-surfaced capacitively coupled electrodes at the O1 and O2 sites in a normal baseball cap for SSVEP detection and at the Oz, Cz, T7, and T8 sites for ASSR detection. All signals were recorded with a reference electrode at A2 and a grounding electrode at FpZ. The reference and grounding electrodes were not active capacitive electrodes but passive dry electrodes that did not require conduction gel or paste. Signals were measured through hair and transmitted through a hardware module composed of a high-pass filter (HPF), a low-pass filter (LPF), a 60 Hz notch filter, and an amplifier with a gain of 10,000. The HPF and LPF were used to reduce fluctuation and for antialiasing, respectively, and were designed as 4^th^-order Butterworth filters from 0.05 to 30 Hz. EEGs were digitized at a 512 Hz sampling rate using an analog-to-digital converter (NI-DAQ Pad 6015, National Instruments Co., TX, USA) and recorded using a Matlab data acquisition toolbox (Matlab2008b, Mathworks, Inc., Natick, MA, USA).

**Figure 4:**
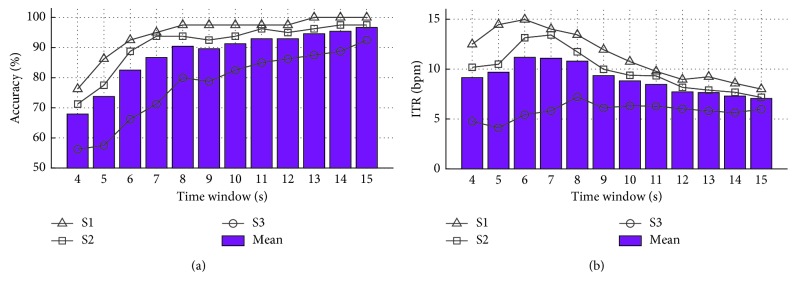
Offline AM-SSVEP analysis: (a) classification accuracy and (b) ITR for each participant with respect to different time window sizes. Bar graph indicates accuracy and ITR averaged over three subjects.

**Figure 5:**
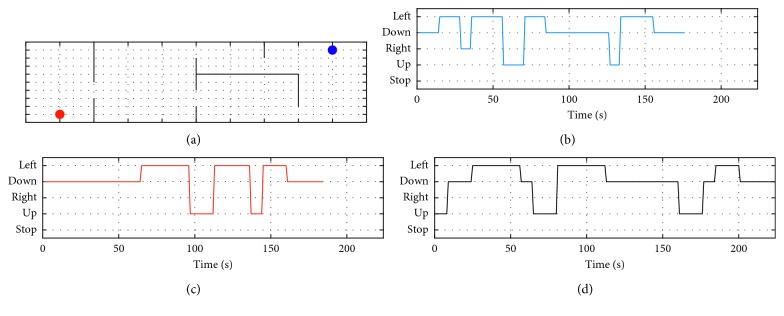
Graphical representation of online AM-SSVEP BCI task: (a) 2D maze in which a cursor (blue dot) can be moved along four possible directions to the target position (red dot); (b–d) movement map that each participant actually performed using the AM-SSVEP BCI.

For the SSVEP-based ABCI application, four visual stimuli were positioned around an LCD monitor in two LED arrays (SMD 5050-3, Korea) with a diffusion film. Four targets (left, up, right, and down) flickered in an amplitude-modulated sinusoidal wave with different combinations of carrier and modulation frequency. The AM stimulus was digitally generated in eight bits at 1000 Hz using a microcontroller unit (ATmega128, Atmel, USA) and then converted into an analog signal again to operate the LEDs using a digital-to-analog converter (LTC1657CN, Texas Instrument, USA). From equation (1), the spectrum of *S*(*t*) has a peak frequency of *f*_c _+ *f*_m_ and *f*_c _− *f*_m_. In this study, the *f*_cs_ were high frequencies (50 and 51 Hz) to reduce eye fatigue, and the *f*_ms_ were low frequencies near the *α*-band (9–12 Hz) to achieve a large SSVEP amplitude and allow high-frequency stimuli carrying low-frequency information to be generated. Figures 2(a)–2(c) provide examples of *c*(*t*), *m*(*t*), and *S*(*t*) and their spectra. When *f*_c_ and fm were 50 and 11 Hz, respectively, spectral peaks of *S*(*t*) appeared at 39 Hz (=(50 − 11) Hz) and 61 Hz (=(50 + 11) Hz). All subjects performed an offline experiment first to determine the optimal EEG analysis time window size when measuring AM-SSVEP after exposure to only visual stimulation without an actual BCI application. Using programmed auditory instructions, the subjects were asked to focus on one of the four targets for 15 s. Each run contained 40 trials, and 2 runs with a 10 min break between them constituted the offline experiment. Each target was attended equally, 10 times per run. Time window sizes from 5 to 10 s with 1 s resolution were tested to investigate time-sensitive changes in the AM-SSVEP-based ABCI system performance. EEG frequency recognition under AM-SSVEP was performed using a canonical correlation analysis (CCA) to find the maximal correlation between the EEG electrode signal and signals from a matrix of templates corresponding to the AM-SSVEP stimulus frequencies, *f*_c _+ *f*_m_ and *f*_c _− *f*_m_.  Figure 4 shows the classification accuracies and ITR for each participant with respect to different time window sizes. Based on the trade-off relationship between the time window size/accuracy and ITR, we selected 7 or 8 s as the optimal analysis window size. Then, the online experiments were conducted using electrode-equipped caps. The task for the online experiment was a simple 2D maze in which a cursor (blue dot) could be moved in four possible directions toward a target position (red dot) (Figure 5(a)). The movement direction was determined by the SSVEP responses from focusing on one of four targets, which were oriented in the four cardinal directions. Figures 5(b)–5(d) illustrate the movement map that each participant actually performed using AM-SSVEP BCI, and Table 2 summarizes the online experimental results for each subject. Efficiency (EFF) was defined as the minimum number of commands necessary to reach the target position divided by the number of commands issued during the run [56]. All subjects successfully completed the task, and average accuracy (ACC), EFF, and ITR were 86.07%, 79.46%, and 8.78 bits per minute, respectively. The AM stimuli were perceived as less annoying than conventional visual stimuli, but we found no other differences between the experimental conditions. The feasibility of the AM-SSVEP BCI using foam-surfaced capacitive EEG electrodes for successful BCI performance and low eye fatigue was confirmed using our current offline and online experiments. A demonstration video can be found at https://youtu.be/YYbHM4HDTeg.

**Figure 6:**
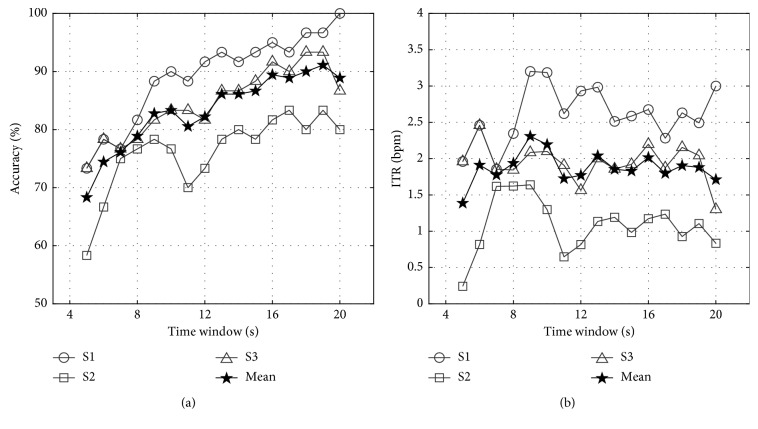
Offline, natural-sound ASSR analysis: (a) classification accuracy and (b) ITR for each participant with respect to different time window sizes. The thick line indicates accuracy and ITR averaged over three subjects.

For the ASSR-based ABCI, 37 and 42 Hz were selected as the message frequencies because the optimal modulation frequency for ASSR has been reported to be around 40 Hz. A water stream and insect sound were chosen as alternatives to the conventional pure tone burst to provide a natural and pleasant sensation. Also, subjects could easily distinguish the different sound streams. The water stream, amplitude-modulated with a 37 Hz message frequency, was presented in the left sound field, and the insect sound, amplitude-modulated with a 42 Hz message frequency, was presented in the right sound field. Each of our three participants sat in a comfortable chair in front of a pair of commercial speakers (BR-2100S, Britz International, Paju, Korea) while wearing a cap containing the foam-surfaced capacitive electrodes. First, subjects performed an offline experiment. Following programmed visual and auditory instructions provided right before the onset of each stimulus, each participant was asked to concentrate on one of the stimuli (L or R) for 20 s. This process was repeated 50 times, and ten-fold cross validation was applied to compare our results with the performance reported in [35], which used the same EEG system but a pure-tone sinusoidal carrier sound for an ASSR BCI. We calculated the frequency spectra using a nonparametric periodogram method with a 1 s sliding time window and 50% overlap. The spectral density of each electrode over the stimulus message frequency ±1 Hz range was extracted from the averaged frequency spectra and fed into a linear discriminant analysis classifier as a feature vector. Classification accuracy and ITR with respect to the time window size are presented in Figure 6. The average value (bold line) showed a pattern similar to that in [35]: accuracy increased linearly with respect to window size. The online experiments were performed after allowing the subjects to have a brief rest. During the resting time, participants took off the electrode cap briefly, and then they put it back on right before starting the online experiment. An analysis window size of 14 s, derived from [35], was used in the online experiments. Ten trials of selective attention to either the left or right stimulus were performed during the online experiments, and the results are shown in Table 3: the number of correct decisions (NUM) 8/10, specificity (SPEC) 0.82, sensitivity (SENS) 0.79, and ITR 1.33 bits per minute. SPEC and SENS were calculated by assuming positive to be left (L) and negative to be right (R). These results are comparable to previously reported results from a study of the same EEG system and ASSR-based technique using conventional pure tone carrier sounds (NUM 7.2/10, SPEC 0.64, SENS 0.76, ITR 0.7 bits per minute). A video demonstration can be found at https://youtu.be/uPF_MjNEefA.

## 5. Discussion and Conclusion

This paper has focused on the challenges faced when moving from BCI systems designed for experimental use in laboratory settings to those intended for use in real-world environments. We have discussed the problems with EEG sensing technologies and new BCI paradigms and explored representative methods for handling laboratory, more realistic, or real-world settings.

We also demonstrated results from an ABCI system that uses foam-surfaced capacitive EEG electrodes with the AM-SSVEP and natural ASSR paradigms. In our AM-SSVEP experiments, we found that classification accuracy was increased with the analysis time window size. However, compared with Chang's result derived using conventional Ag/AgCl electrodes [46], an increased time window was required to sufficiently reject the extra noise seen with capacitive measurements. EEG signals longer than 4 s were suitable for reliable AM-SSVEP BCIs in Chang's study, whereas 8 s were required for the capacitive AM-SSVEP BCI in this study. In our experience, a time window of 8 s is the longest used to determine a single command by SSVEP response. That long time window is required to capacitively measure AM-SSVEP because of the low signal-to-noise ratio. A time window of 6 s was enough for a successful conventional SSVEP-based spelling task with capacitive EEG measurement performed by Baek et al. in 2013 [35]. For online BCI, a direct comparison between the online BCI performance in the current study and Chang et al.'s result [46] cannot be made due to the duration of processing, the classified command, and our use of a different task with a different number of possible selections. Chang et al. stored EEG data in a 4 s data buffer every 0.5 s. The existing data were shifted, removing the initial 0.5 s of data to generate a new 4 s segment. Then, the AM-SSVEP was recognized within the 4 s EEG signal by using the CCA method every 0.5 s. If four consecutive temporal decisions were the same, the corresponding decision was selected as the final decision. In addition, a customized frequency was combined with the best performance of each subject to create a CCA reference signal for frequency recognition. In our experiment, decisions were made at every fixed time window without any data shifting or window sliding. Also, the frequency of the reference signal in the CCA was not optimized for each subject. We chose those parameters for convenience in the experimental setting. Nevertheless, all the participants succeeded in carrying out the cursor navigation task with relative ease. All of the subjects indicated that they preferred working with the AM-SSVEP approach despite the lower accuracy rates and reduced speed of operation because the flickering was less tiring and required less effort from the eyes. The ASSR-based BCI paradigm was used in 2011 by Kim et al. with conventional EEG electrodes and auditory stimuli generated using periodic amplitude-modulated and pure sinusoidal tones [57]. They implemented a pilot online ASSR-based BCI and tested it with one subject. Their experimental result showed a classification accuracy of 71.4%. Our previous study, performed in 2013 by Baek et al., used the same auditory stimulation but foam-surfaced capacitive electrodes instead of gel-based electrodes for the EEG sensing; we had an average accuracy of 72% for 5 subjects [35]. Compared with previous studies, we found a fair average classification accuracy of 80% for 3 participants under ABCI conditions in this study, and we acquired the EEG signals over hair using foam-surfaced capacitive electrodes and natural sound.

Although the number of subjects in the current demonstrations is too small to be representative of the general population, our results are in general agreement with published values for healthy adults, and we suggest that our demonstration offers sufficient power to assess the feasibility of ABCI applications. However, our results represent just a small sample of the broad future potential of ABCI technologies. As BCIs become more popular with different user groups, including healthy people, their increasing commercial possibilities will likely encourage new applied research efforts that will make BCIs even more practical. Consumer demand for reduced cost, increased performance, and greater flexibility and robustness could contribute substantially to making BCIs mainstream tools. The development of ABCIs requires clear validation of their real-life value in terms of efficacy, practicality, and impact on quality of life. Future BCI systems should (1) be comfortable, convenient, and offer aesthetically acceptable mountings, (2) be easy to set up, (3) function for many hours without maintenance, (4) perform well in all environments, (5) operate by telemetry instead of requiring wiring, and (6) interface easily with a wide range of applications. Before the results of ongoing and planned research efforts for ABCI become available, BCIs using various methods remain a fascinating research toy. If the intensive research into various aspects of ABCI continues to increase exponentially, as it has done recently, BCI systems could become routine clinical, assistive, and commercial tools in the not-too-distant future.

## Figures and Tables

**Table 1:** Summary of representative dry EEG electrodes.

Type	Fabrication	Flexibility	Ref.	BCI application	Strengths	Drawbacks
Electrode finger	Copper pin	Stiff	[29]	SSVEP	(i) Simple manufacturing procedure for mass production	Subjects felt pain or discomfort from the pressure
					(ii) Small size (*d* = 15 mm) for good spatial resolution	
	Spring loaded pin coated with gold	Soft	[36]	N/A	(i) Small size (*d* = 15 mm) for the good spatial resolution	Equipment required for electrode fixation
					(ii) High level of geometric conformity between the sensor and the scalp surface	
	Flexible polymer pin	Soft	[37]	N/A	Subjects reported that they were more comfortable than the conventional EEG system	Slight erythema was found after 10–35 h, but it faded rapidly after the electrode was removed

Toothbrush	Silver-coated bristles	Soft	[30]	(i) Motor imagery	Better comfort than wet or pin-based electrodes	(i) Requires multistep preparations
				(ii) P300		(ii) Some subjects felt prickling sensation
				(iii) N100		

Reverse-curve-arch-shaped	Sterling silver using 3D printer	Stiff	[31]	N/A	Maximized contact area and dispersed pressure	(i) Obtrusive wiring interfaces.
						(ii) Equipment required for electrode fixation

Epidermal (tattoo)	Microfabrication with polyimide	Soft	[33]	(i) SSVEP	(i) Soft, stretchable, and lightweight	(i) Cannot be applied to hairy scalp
				(ii) P300	(ii) Conformal contact, adequate adhesion	(ii) Sensor design problem for people who do not want stick-on, temporary tattoos

Capacitive	CNT/aPDMS	Stiff	[32]	(i) SSVEP	(i) Electrode could be autonomously attached to the scalp without the need for additional equipment.	Require multistep preparations, obtrusive wiring interfaces
				(ii) N100	(ii) Small size (*d* = 6 mm) for good spatial resolution	
	Custom-integrated AFE	Stiff	[40, 41]	SSVEP	No need for external G- or T-ohm for biasing network	(i) Weak for motion artifacts
						(ii) Poor coupling interface through dry hair
	Polymer foam surfaced	Soft	[34, 35]	(i) SSVEP	(i) EEG measurement through hair	(i) Weak for motion artifacts
				(ii) ASSR	(ii) Comfortable for users	(ii) Low spatial resolution due to relatively large size (*r* = 36 mm)
					(iii) Higher signal quality than with rigid capacitive electrodes	

**Table 2:** Results of the AM-SSVEP-based online BCI experiments (ACC: accuracy in %, ITR: information transfer rate in bit·min^−1^, LPM: letters per minute in letter min^−1^, and EFF: efficiency in %).

Sub.	Time window	Output path (error underlined)	ACC (%)	EFF (%)	ITR (bits/min)
S1	7 s	DDDDDDDDLLLLUULLLULLLDDD	84	80	8.01
S2	8 s	DDLLRLLLUULLDDDDDDULLDDD	95.65	86.96	12
S3	8 s	UDDLLLLDUULLLLDDDDDDUUDLLDDD	78.57	71.43	6.34
Mean			86.07	79.46	8.78

**Table 3:** Results from natural-sound ASSR-based online BCI experiments (NUM: number of correct classifications per total number of trials, SEPC: specificity, SENS: sensitivity, and ITR: information transfer rate in bit·min^−1^). Inaccurate classifications are underlined.

Sub.	Time window	Task	Classification results	NUM (correct/total)	SPEC	SENS	ITR (bits/min)
S1	14 s	LLRLRLRLRR	LLRRRLRLRR	9/10	1.0	0.83	2.28
S2	14 s	LRRLRRLRLL	RRRLRLLRLR	7/10	0.67	0.75	0.51
S3	14 s	RRRLLRLLRL	RRRLRLLLRL	8/10	0.8	0.8	1.19
Mean				8/10	0.82	0.79	1.33
